# Regulation of p53^wt^ glioma cell proliferation by androgen receptor-mediated inhibition of small VCP/p97-interacting protein expression

**DOI:** 10.18632/oncotarget.15509

**Published:** 2017-02-19

**Authors:** Dejun Bao, Chuandong Cheng, Xiaoqiang Lan, Rong Xing, Zhuo Chen, Hua Zhao, Junyan Sun, Yang Wang, Chaoshi Niu, Bo Zhang, Shengyun Fang

**Affiliations:** ^1^ Department of Pathophysiology, School of Basic Medical Science, Dalian Medical University, Dalian, China; ^2^ Department of Neurosurgery, Anhui Provincial Hospital Affiliated to Anhui Medical University, Hefei, China; ^3^ Anhui Provincial Stereotactic Neurosurgical Institute, Hefei, China; ^4^ Anhui Province Key Laboratory of Brain Function and Brain Disease, Hefei, China; ^5^ Department of Neurosurgery, 2nd Hospital of Dalian Medical University, Dalian, China; ^6^ Center for Biomedical Engineering and Technology, Department of Physiology, Department of Biochemistry and Molecular Biology, University of Maryland, School of Medicine, Baltimore, Maryland, USA; ^7^ Anhui Provincial Cancer Hospital (West Branch of Anhui Provincial Hospital), Hefei, China; ^8^ Department of Clinical Laboratory, Cancer Hospital, Chinese Academy of Science, Hefei, China

**Keywords:** AR signaling, SVIP, cell proliferation, serum testosterone, p53

## Abstract

The incidence of glioma in men is higher than that in women; however, little is known about the expression and basic function of the androgen receptor (AR) in gliomas. AR inhibited the small VCP/p97-interacting protein (SVIP) on the transcriptional level was previously reported. The present study shows that the protein level of AR is highly expressed in cell lines of the nervous system. Moreover, the AR expression is increased while SVIP expression is decreased in tumor tissue of glioma patients, which is in agreement with the progressing WHO grades. A statistically significant increase in serum testosterone level of glioma patients compared with that of non-cancer patients was also detected. Furthermore, it has been proved that SVIP is down-regulated as well as AR is up-regulated in glioma cell lines with R1881 treatment. Interestingly, the depletion of SVIP using siRNA facilitated cell proliferation and decreased p53 expression. In addition, overexpression of SVIP increased cell death only in p53^wt^ cell lines. Moreover, U87MG cells, p53^wt^ cell line was susceptible to AR antagonists *in vitro* and *in vivo*. The current study provides insight into the biological role of AR in suppressing SVIP and p53 and promoting the progression of glioma as well as the clinical treatment of glioma patients.

## INTRODUCTION

A glioma arises from glial cells. Gliomas constitute approximately 30% of all brain and central nervous system tumors and 80% of all malignant brain tumors and are rarely curable [1]. According to a report in 2008, out of 10000 Americans diagnosed each year with malignant gliomas, approximately half were alive one year after diagnosis, and 25% after two years [2], whereas glioblastoma multiform (GBM) has a worse prognosis [3]. The exact causes of gliomas are still unclear. However, an epidemiological survey has demonstrated that the incidence of glioma in men is about 1.5 to 2 fold of that in women, regardless of etiologies [4, 5], suggesting a sex-related discrepancy. With respect to the differences in sex hormones between the two genders, the androgen/testosterone level in the serum may be underestimated in the pathogenesis of gliomas.

Androgens play a crucial role in gender differentiation, development and expression of the male phenotype, and exert their biological effects through interaction with the androgen receptor (AR) [6]. Upon such interaction, AR undergoes a conformational change that allows nuclear translocation, DNA binding and regulation of the target gene [7, 8]. Previous studies have shown that AR is overexpressed in some human malignancies, such as prostate cancer, breast cancer, hepatocellular carcinoma, and bladder cancer [9–11]. However, till date, only one study has examined AR signaling in GBM [12], although a limited number of tumor tissues specimens were used, and no statistical analysis was performed. On the other hand, the serum testosterone level is still unknown in glioma patients.

Two anti-androgens, ARN-509 and MDV3100 inhibiting AR nuclear translocation and AR binding to androgen response elements [13, 14], for prostate cancer treatment have been demonstrated to significantly improve survival in men with metastatic castration-resistant prostate cancer (CRPC) in several clinical trials [15]. However, only the latter one was approved by the FDA in 2012.

It has been reported that in response to a physiological concentration of metabolically stable androgen (R1881), the small VCP/p97-interacting protein (SVIP) expression could be down-regulated by AR on a transcriptional level in prostate cancer cell line LNCaP [16]; however, the phenomenon has not been established on a protein level till date. SVIP is now known as a bi-functional protein. It serves as an endogenous inhibitor of the endoplasmic reticulum (ER)-associated degradation (ERAD) [17]. On the other hand, SVIP induces localization of VCP/ p97 to the plasma and lysosomal membranes and regulates autophagy [18]. The tumor suppressor p53 regulates the cell cycle and is commonly inactivated in various types of cancer. Thus, p53 mutations lead to genomic instability and inhibit apoptosis. The occurrence of p53 mutations in primary GBMs is about 10% [3]. Herein, we investigate the variable sensitivities between wildtype and mutant p53 glioblastoma cell lines to androgen receptor agonist or antagonists.

Therefore, the present study aimed to demonstrate that AR signaling and serum T level are related to glioma and provided *in vitro* and *in vivo* xenograft evidence to support AR and SVIP as new targets for p53^wt^ gliomas.

## RESULTS

### Androgen receptor is highly expressed in glioma and neuroblastoma cells

Expression of AR in 11 cell lines was analyzed by Western blot assay (Supplementary Figure 1A). The result indicated that AR was highly expressed in neuroblastoma cell lines, Neuro2A, and SH-SY5Y, as well as prostate cancer cell line LNCaP, glioma cell lines, U87MG and U251MG. However, compared with the above cell lines, little AR was observed in cervical cancer cell line HeLa, colon cancer cell lines, bladder cancer cell line BIU-87, and AR-independent prostate cancer cell line PC-3 (Supplementary Figure 1A). Although many neuronal types are known to express sex steroid receptors [19, 21], we assessed the expression pattern of AR in normal mouse and rat brain tissue by IHC (Supplementary Figure 1B) and IF (Supplementary Figure 1C). In accordance with the findings, almost all the neurons, although from different brain regions, were AR-immunoreactive (Supplementary Figure 1B, 1C). However, the glial cells, astrocytes, microglia, and oligodendrocytes marked by anti-GFAP, integrin-αM, and CNP antibody, respectively, were negatively stained (Supplementary Figure 1C).

### High serum testosterone level in glioma patients

The serum testosterone (T) levels in glioma patients, benign brain tumor patients and normal controls, as well as the comparison of the serum testosterone of glioma patients among age groups and WHO grades, are shown in Table 1. The average serum testosterone level was significantly higher in glioma group compared with the control group (*P* < 0.001) and benign brain tumor group (*P* < 0.001). Moreover, the serum testosterone level was remarkably higher in glioma patients of age 30, 50 years as compared to another age group (*P* < 0.001), irrespective of the gender. Furthermore, the serum testosterone levels were not significantly altered in different WHO grades both in male (*P* = 0.373) and female (*P* = 0.954) glioma patients, suggesting that increased serum testosterone level in glioma patients not be a result of tumor progression. Instead, the T level may rise before the tumor progress. We further analyzed the significance of serum testosterone level differences among age groups in glioma patients, benign brain tumor group, and normal control group (Table 2). Glioma patients over 30 years of age have significantly higher serum testosterone level than benign brain tumor or normal control group in the same age range.

**Table 1:** Serum testosterone (T) level in patients of control group, benign brain tumor group, and glioma group, and comparison of clinical characteristics (X ± SD)

	Male			Female		
	*n*	T (ng ml^−1^)	*P*-value^a^	n	T (ng ml^−1^)	*P*-value
Patient type			< 0.001			< 0.001
Normal control	42	9.47 ± 3.70		76	0.83±0.45	
Benign Brain tumor	64	11.40 ± 4.60		112	0.80±0.58	
Glioma	122	16.49 ± 3.98		67	3.69±1.99	
Age (years)			< 0.001			< 0.001
< 30	11	12.69 ± 2.31		7	3.07±1.41	
30–50	51	18.59 ± 3.26		35	5.02±1.73	
> 50	60	15.40 ± 3.85		25	2.11±0.69	
WHO grade			0.373			0.954
I	7	15.25 ± 2.65		5	3.24±1.54	
II	36	15.79 ± 2.79		13	3.75±2.01	
III	30	16.56 ± 3.69		33	3.68±2.08	
IV	49	17.15 ± 4.91		16	3.83±2.06	

a, *P*-value was estimated using one-way ANOVA; T, serum testosterone concentration; WHO, World Health Organization.

**Table 2:** The significance of serum testosterone (T) level differences among age groups in patients with glioma, benign brain tumor, and normal control group

Age group (years)		Glioma		Benign brain tumor		Normal control	
		*n*	*P*-value^a^	*n*	*P*-value^b^	*n*	*P*-value^c^
< 30	Male	11	0.001	4	–	0	–
	Female	7	0.1	12	–	0	–
30–50	Male	51	< 0.001	32	0.015	12	< 0.001
	Female	35	< 0.001	51	1.000	23	< 0.001
> 50	Male	60	< 0.001	28	1.000	8	0.003
	Female	25	< 0.001	49	0.001	53	< 0.001

a, glioma compared with benign tumor; b, benign tumor compared with normal control; c, glioma compared with normal control. *P*-value was estimated using Bonferroni's Post-Hoc test. The mean difference is significant at 0.05 level.

### AR expression is increased, but SVIP expression is reduced in glioma tissue samples

**Figure 1:**
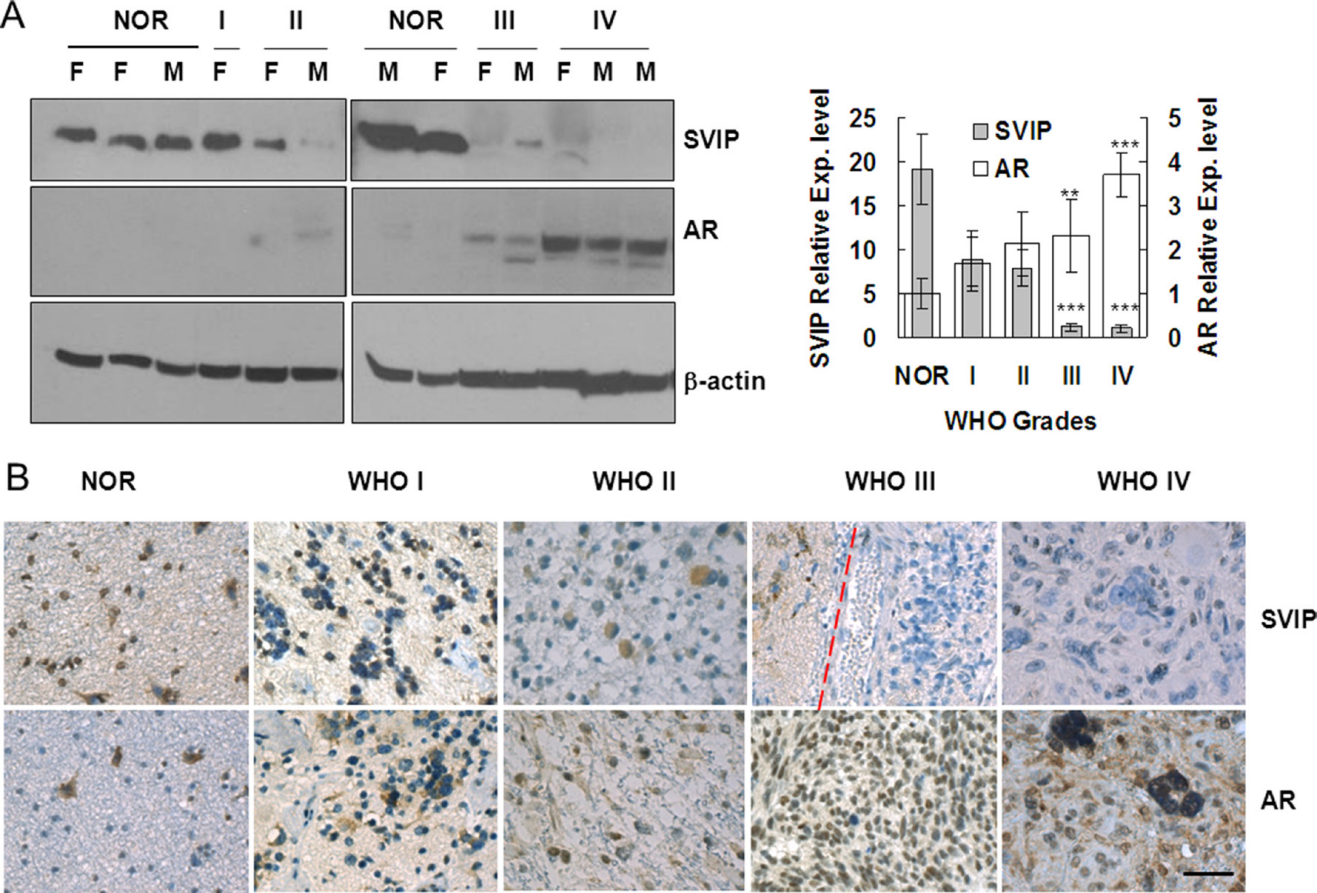
AR expression is increased, but SVIP expression is reduced in glioma samples compared with normal brain tissues Western blotting assay (**A**) and immunohistochemistry staining (**B**) of 73 specimens, including 12 non-cancer patient samples (referred to as NOR subsequently). (A) F, female patient; M, male patient. β-actin was used as a loading control. Error bar represents ± SD, **P < 0.01; ***P < 0.001, WHO III & IV compared with NOR. (B) IHC staining of AR and SVIP in normal and glioma tissues. NOR, trauma; WHO I, subependymal astrocytoma; WHO II, ependymoma; WHO III, astroglioma; WHO IV, glioblastoma, scale bar = 50 μm. In the specimen of WHO III, peritumoral region (left) and tumor region (right) are separated by a dashed red line.

73 specimens, including 12 non-cancer patient tissue samples (NC), were subjected to Western blot assay and immunohistochemistry staining. It has been previously reported that SVIP is inhibited by AR [16]. Hence, we examined SVIP and AR expression in these 73 samples. A 95% downregulation of SVIP expression, from NC to high-grade gliomas (WHO III and IV) was observed (Figure 1A); consecutively, the AR expression increased as the tumor grade progressed, irrespective of the gender (Figure 1A). Especially, WHO IV tumor tissues showed a remarkable level of AR expression but were nearly undetectable in NC. Consistent with the Western blot result, the immunohistochemistry staining showed lower expression of SVIP in high-grade gliomas than in the NC, and a reverse trend of AR expression in the nucleus was observed (Figure 1B). The occurrence of AR immunoreactive specimens in grade I to IV was 25%, 73.3%, 91.3%, and 96.3%, respectively (Table 3). The relative expression level of AR closely correlated with the pathological grades (F = 14.369, *P* < 0.001). Interestingly, the cells located around the blood vessels in the high-grade tumor tissues expressed AR at an extraordinarily high level (Supplementary Figure 2). All these results illustrated that the decreased SVIP expression, as well as increased AR expression, in glioma tissues correlated with gliomas progressing from low to high grades.

**Table 3:** The correlation between the pathological grade and the expression of AR in gliomas tissues (X ± SD)

Pathological Grade	The expression of AR				
	–	+	++	+++	Immunopositive ratio (%)
Normal brain tissue (*n* = 12)	12	0	0	0	0
WHO Grade I (*n* = 8)	6	2	0	0	6.83 ± 8.38
WHO Grade II (*n* = 15)	4	6	5	0	13.91 ± 10.99
WHO Grade III (*n* = 23)	2	3	11	7	44.32 ± 27.33
WHO Grade IV (*n* = 27)	1	4	6	16	61.52 ± 27.07

–, negative staining; +, weak positive staining; ++, moderate positive staining; +++, strong positive staining.

Immunopositive ratio = (number of positive cells/1000) × 100%.

### AR is upregulated, and SVIP is downregulated in glioma cell lines with R1881 treatment

**Figure 2:**
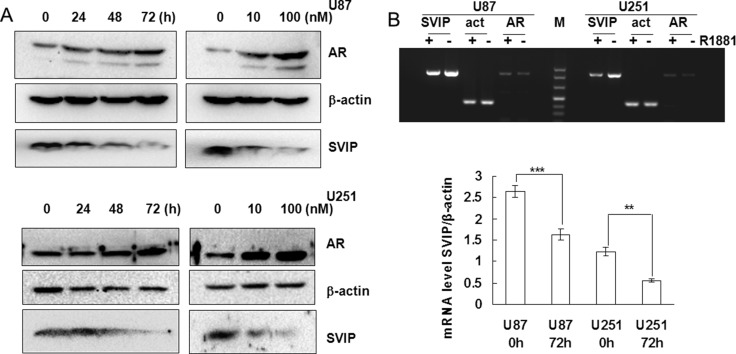
AR inhibits SVIP expression on transcriptional level in glioma cell lines (**A**) R1881 treatment enhanced AR stability but decreased SVIP expression in U87 and U251 cell lines, which is associated with the time and dosage of R1881 treatment. β-actin was used as a loading control. (**B**) Semi-quantitative PCR analysis of SVIP, AR, and β-actin mRNA level after 72 h treatment of R1881 in U87 and U251 cells. Bar graph indicates the bands’ intensity of SVIP mRNA at 0 h (72 h DMSO treatment) and 72 h of R1881 treatment, calibrated with β-actin RNA level. *n* = 3, **P < 0.01;***P < 0.001.

AR, a transcriptional regulator, was translocated in the nuclear region with R1881 treatment, as assessed by immunofluorescence in U87 cells (Supplementary Figure 3A). Unlike advanced prostate cancer cells, androgen receptor splice variant 7 (AR-V7) cannot be detected in U87 or U251 cells (Supplementary Figure 3B) suggesting glioma cells may sensitive to androgen receptor agonist or antagonist. Downregulation of SVIP protein was detected by Western blotting when U87 or U251 cells were treated with R1881, a synthetic androgen, in a time and concentration-dependent manner (Figure 2A). Consistent with WB result, SVIP RNA level analyzed by PCR assay was also distinctly decreased after 72 h post 10 nM R1881 treatment (Figure 2B). Taken together, these results indicated that SVIP was downregulated by AR and also explained that its expression was low in high-grade glioma tissue samples.

### SVIP inhibits proliferation of HeLa and U87 cells but had no effect on that of U251 or SKBR-3 cells

**Figure 3:**
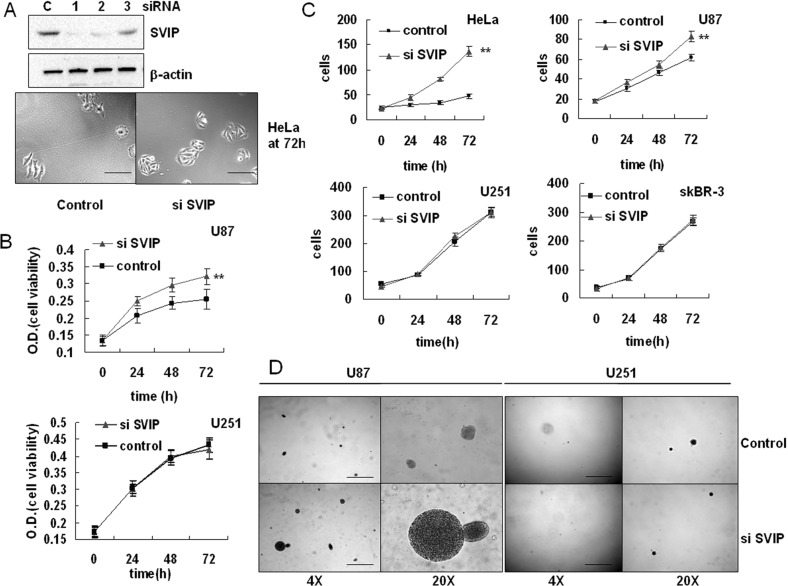
Depletion of SVIP by siRNA leads to increased proliferation of U87 but not U251 cells (**A**) HeLa cells transiently transfected with the control siRNA (**C**) or three individual SVIP siRNAs (siRNA #1, 2 and 3) were subjected to western blotting analysis of SVIP and actin (as a loading control), siRNA #1 was used thereafter for SVIP knockdown. siSVIP leads to increased cell number as well as small size of HeLa cells compared with the control, scale bar = 50 μm. (**B**) siSVIP led to increased proliferation of U87 cells, but had no impact on U251 cells, analyzed by MTT assay. Error bar indicates standard deviation, *n* = 4, **P < 0.01. (**C**) siSVIP leads to greater U87 and HeLa cell proliferation, but had no effect on U251 and SKBR-3 cell proliferation, analyzed by cell count. The number of cells was calculated in 5 random view fields/well in a 6-well plate, *n* = 3. Error bar indicates standard deviation, **P < 0.01. (**D**) Soft agar assay of anchorage-independent growth on U87 and U251 cell line transfected with SVIP siRNA or siRNA control, scale bar = 2 mm. Data are representative of three individual experiments.

The effect of SVIP knockdown by siRNA on cell proliferation was evaluated by MTT assay. The endogenous SVIP expression was knocked down in GBM U87 and U251 cells by RNAi. Compared with the control group, U87 cells transfected with siSVIP showed increased viability (Figure 3B). Nevertheless, we also found that the average size of HeLa cells appeared smaller than the control (Figure 3A) when SVIP was depleted. Thus, we speculated that the increased viability of SVIP depleted cells was due to increased cell number or elevated cellular metabolism. Thus, a cell count method was applied on HeLa, U87, U251, and SKBR-3 cell lines. Both the p53^wt^ cells lines showed rapid growth with SVIP knockdown (Figure 3C). However, U251 cells did not exhibit any response to endogenous SVIP depletion (Figures 3B and 3C), whereas breast cancer cell line SKBR-3 (p53 mutant) did not show altered viability (Figure 3C). Furthermore, to investigate the anchorage-independent growth of tumor cells, a soft agar colony formation assay was performed. As anticipated, after 16 days, SVIP depletion resulted in U87 cell colony growth. Thus, we concluded that the depletion of SVIP might increase cell proliferation in GBM U87 cells but not in the p53 mutant U251 cell line.

### SVIP depletion decreased p53 expression, and overexpression increased cell death of U87 cells

**Figure 4:**
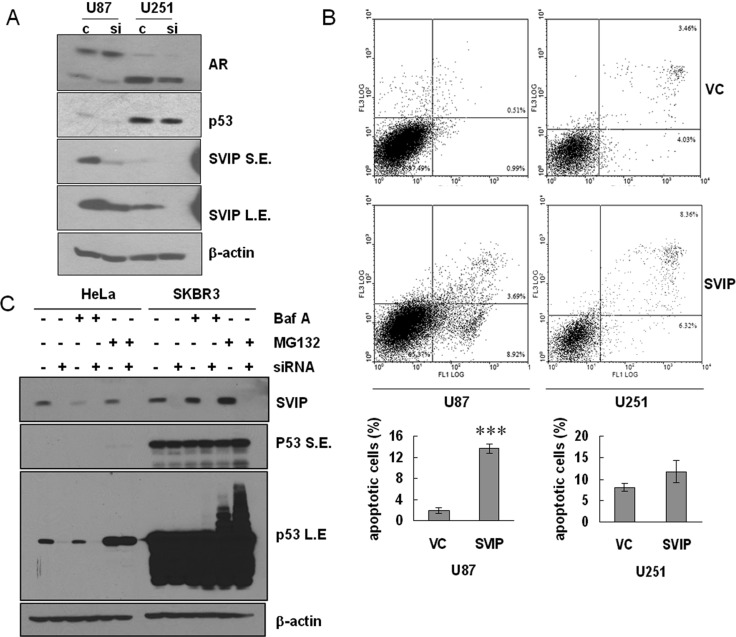
Depletion of SVIP by siRNA causes declined protein level of p53^wt^ and overexpression of SVIP leads to apoptosis in U87 cells (**A**) Depletion of SVIP by siRNA causes declined protein level of p53 in U87MG but not in U251MG cells. S.E., short exposure time. L.E., long exposure time. β-actin was used as a loading control. (**B**) SVIP overexpression increased apoptosis as compared to cells transfected with vector control (VC). 6 × 10^5^ U87 cells or 3 × 10^5^ U251 cells in a 6-well plate were transfected with 1 μg SVIP expressing plasmid or vector control (VC), respectively, using Lipofectamine 2000. 24 h after transfection, apoptotic cells were determined by Annexin V (X-axis)/Propidium Iodide (Y-axis) staining followed by flow cytometry. Error bar indicates standard deviation, *n* = 3, ***P < 0.01. (C and D) SVIP promotes p53 protein expression on a post-transcriptional level in HeLa and SKBR-3 cell lines. (**C**) Western blotting assay of p53 protein level shows downregulation by siSVIP. HeLa and SKBR-3 cells were treated with BFA (10 ng ml^−1^) to inhibit autophagy or MG132 (10 μM) to inhibit proteasome for 2 h and then processed for immunoblotting for the indicated proteins. β-actin was used as a loading control. S.E., short exposure; L.E., long exposure.

Intriguingly, downregulation of p53 was detected by Western blotting assay when SVIP was knocked down in U87 cells with siRNA (Figure 4A). It was previously reported that AR and p53 can inhibit one another mutually on a transcriptional level [22, 23]. When p53 decreased in U87 SVIP knockdown cells, its inhibition effect on AR gene transcription became attenuated. Thus, p53 reduction resulted in increased AR expression (Figure 4A). Additionally, we examined if the overexpression of SVIP could promote apoptosis in GBM cells. The proportion of apoptotic cells transfected with SVIP expressing plasmid was 740% greater than that transfected with vector control (Figure 4B) in U87 cells. On the contrary, U251 cells did not show significant alteration in apoptosis. In order to understand the regulatory mechanism of SVIP on p53, we treated HeLa and SKBR-3 cells, to exclude the feedback effect of AR-p53 mutual suppression, with SVIP siRNA and proteasome inhibitor MG132 or lysosomal inhibitor bafilomycin A1 (Baf A). In HeLa p53 wild type cell line, p53 decreased when SVIP was knocked down with siRNA, and the expression was restored by the proteasome inhibitor MG132, but not a lysosomal inhibitor (Figure 4C). It suggested that SVIP depletion decreased p53 protein expression. Moreover, the RNA analyzed by reverse transcriptional PCR did not display a decreased p53 level upon SVIP depletion in either HeLa or SKBR-3 cells (Supplementary Figure 4A). Taken together, these results indicate that decreased p53 protein expression due to SVIP depletion only occurs in p53 wild-type cells. Moreover, the cell proliferation effect of siSVIP and SVIP-induced apoptosis are both p53 dependent.

### AR antagonists delayed U87 proliferation *in vitro* and *in vivo*

**Figure 5:**
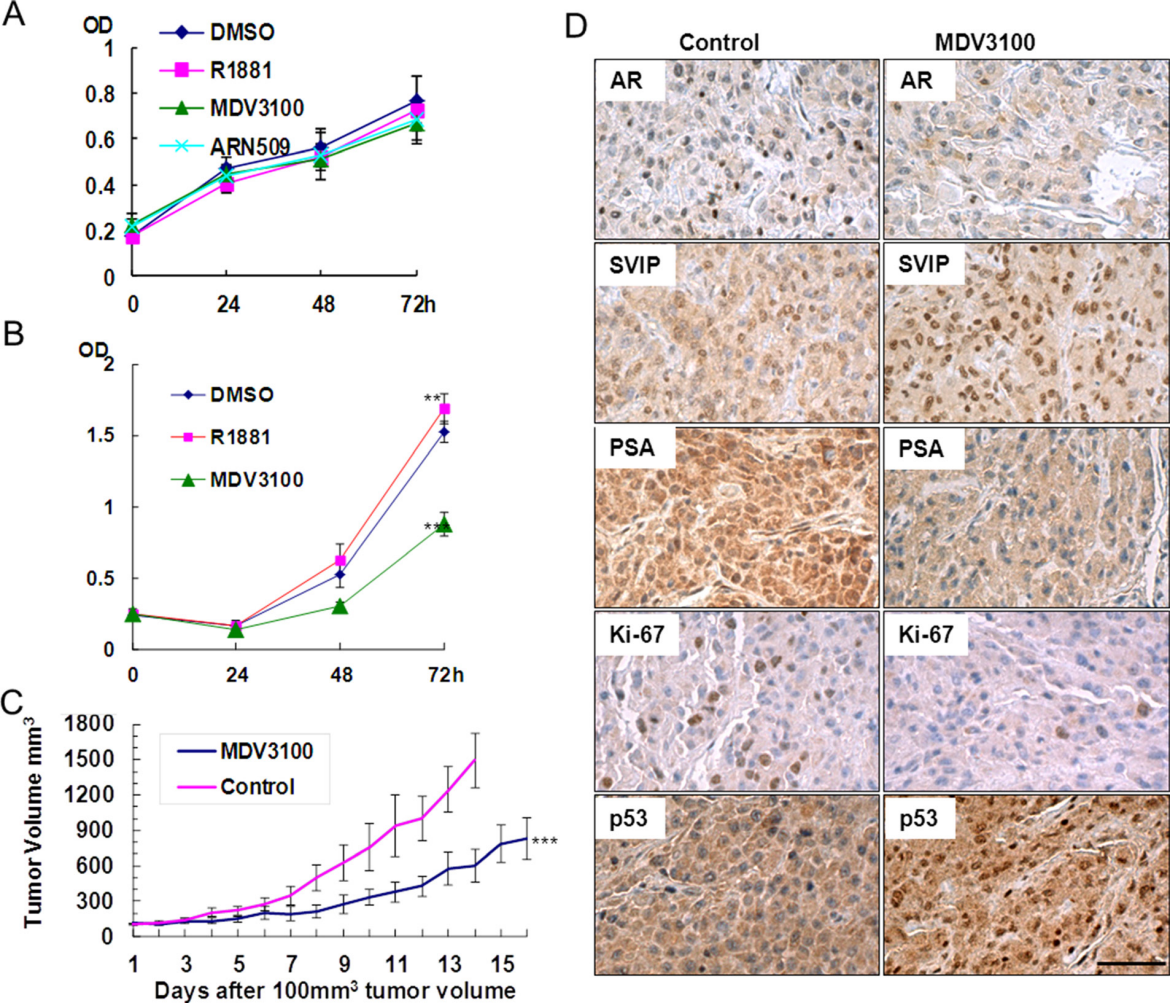
AR antagonist inhibited U87 proliferation *in vitro* and *in vivo*, but it had no effect on U251 (**A**) U251 and (**B**) U87 MTT assay. Cells cultured in phenol red-free DMEM containing 5% (v/v) charcoal stripped fetal bovine serum supplemented with 0.1% (v/v) DMSO or 10 nM R1881 or 10 μM ARN-509 or 10 μM MDV3100 (final concentration), error bar indicates standard deviation, **P < 0.05, ***P < 0.01. (**C**) MDV3100 delayed the growth of the U87 tumor. Nude mice were treated with MDV3100 for 16 days or placebo (control group) for 14 days daily by oral gavages after the tumor volume reached 100 mm^3^ (*n* = 8). Error bar indicates standard error, ***P < 0.01. (**D)** Immunohistochemistry analysis of AR, SVIP, PSA, p53, and Ki-67 performed on paraffin-embedded tumor tissue sections obtained from executed mice after 14 days of placebo or MDV3100 treatment, scale bar = 100 μm.

If the growth of glioma cells relied on AR signaling pathway, it could be delayed with an appropriate AR antagonist and vice versa. Hence, we tested ARN-509 and MDV3100, AR-targeted novel drugs for CRPC patients sharing the same anti-androgen mechanism [24], *in vitro* and *in vivo*. Unlike U87 cells, neither R1881 nor two AR antagonists, MDV3100 and ARN-509, exhibited any effect on proliferation of U251 cell line (Figure 5A). On the other hand, 10 μM MDV3100 can dramatically decrease the growth of U87 cells (Figure 5B). Furthermore, to investigate whether AR antagonist could inhibit glioma growth *in vivo*, flank tumor xenograft model bearing U87 cells was treated with MDV3100 or placebo (vehicle control) by daily oral gavage. The mean tumor volume, which was measured with the caliper directly (Figure 5C) and live animal imaging, in MDV3100 group was obviously smaller compared with that of placebo group after 2 weeks of drug administration. IHC analysis also proved that the activated AR signaling pathway contributes to tumor growth (Figure 5D). Prostate cancer is characterized by elevated levels of prostate-specific antigen (PSA). Interestingly, we observed robust expression of PSA coupled with AR in the control group in contrast to MDV3100 group. Also, we observed elevated SVIP and p53 level in MDV3100 group; that was in agreement with the anti-AR effect of MDV3100 treatment. Moreover, higher Ki67 expression shown in placebo group reflected a high proliferation rate. All the above results demonstrated that the proliferation of glioma U87 cells *in vitro* and *in vivo* was closely related to the AR-SVIP-p53 axis.

## DISCUSSION

The etiology of the primary tumors suggested that the female sex hormones are protective against glioma [25]. Moreover, several studies revealed more cases of glioma among males than females [26, 27]. In addition, animal experiments provided evidence that androgens facilitated the growth of glioblastomas, whereas estrogen increased survival in the animal model of glioblastoma [28, 32]. However, the testosterone level of glioma patients has not been confirmed till date.

In order to investigate whether AR signaling plays a role in the pathogenesis of glioma, firstly we checked the AR protein expression in 11 tumor cell lines, mouse and rat brain tissue. We found that AR expressed in the glioma cell lines, neuroblastoma cells, and neurons but not in glial cells. Hence, the AR expression may be associated with glioma's tumorigenesis in the brain. A critical factor, serum testosterone level of glioma patients, was not fully understood previously. Herein, we demonstrated the serum testosterone concentration of glioma patients higher than non-cancer individuals. Furthermore, the expression level of AR, analyzed by WB and IHC, in tumor tissue was closely correlated with the malignancy of glioma, whereas SVIP protein expression showed an opposite trend. Although it has been reported that SVIP transcription is suppressed by AR at a transcriptional level [16], it has yet not been experimentally established. In the present study, we demonstrated the effect of R1881 treatment on increased AR protein level and decreased protein and mRNA level of SVIP in both U87 and U251 cells, respectively. This was in agreement with that observed in glioma tissues. Moreover, to investigate SVIP's impact on cell proliferation, MTT, soft agar assay as well as cell number count were performed in p53 wild-type cells, U87, and HeLa, as well as U251 and SKBR-3 p53 mutant cells. Considering the p53 genotype of these cell lines, especially the two GBM cell lines, U87 and U251, the result illustrated that p53 wild type cell lines may be responsive to SVIP depletion by siRNA, thereby leading to increased cell proliferation; however, p53 mutant cell lines may not. p53 is a known tumor suppressor that can negatively regulate cell growth and promote apoptosis. Hence, we presumed that increased cell proliferation in SVIP knockdown cells might rely on reduced p53 expression. The overexpression of SVIP promoted apoptosis, and depletion by siRNA led to declined protein level in p53^wt^ cell line but no effect on p53 mRNA. This suggested that SVIP inhibited the cell growth, at least partially, in a p53-dependent manner. Finally, we established that p53^wt^ U87 cells were sensitive to AR antagonist *in vitro* and *in vivo*, but U251 cells were not sensitive to AR antagonist. Based on these results, we propose that AR and SVIP may play major roles in the proliferation of p53^wt^ glioma cells.

Glioma is the most common brain tumor, and the prognosis of patients remains very poor. Currently, temozolomide (TMZ) is the most widely used drug for chemotherapy of glioma patients; however, in most cases, the patients and doctors are far from being satisfied with this approach, due to only slight improvement in prognosis [33]. On the other, gliomas can easily relapse [3]. Different from other tumors, the surgery area of glioma must be strictly restricted to the tumor region to avoid impairment of the normal function of the brain. Gross total resection with a low risk of morbidity (i.e., onco-functional balance) is considered as the goal of surgery [34]. Thus, in the current study, AR and SVIP expression in each grade glioma tissue are mostly compared with normal tissues, instead of peritumoral tissues (Figure 1).

p53 mutations occur more frequently in secondary GBMs (> 60%) than in primary GBMs (~10%) and primary GBM accounts for up to 95% of the GBM cases [3]. In our study, p53 mutant cells presented insensitivity to AR antagonists, such as MDV3100 and ARN-509, suggesting that although all those have been defined as GBM, their genotypes may vary. Importantly, the therapeutic regimens may also vary. For example, p53 genotype analysis should be performed after surgery as the therapeutic regimen may depend on the genotype of p53 rather than simply treated with TMZ or other anti-tumor drugs. We found that SVIP promoted p53 expression on the post-transcriptional level (Figure 4C and Supplementary Figure 4A). Since SVIP is an ERAD inhibitor [17], it can delay the degradation of p53 (Figure 4C and Supplementary Figure 4B). However, the underlying mechanism will be illustrated in our future studies.

The role of AR is widely studied and recognized in prostate cancer. Thus, targeted chemotherapeutics against AR have been developed. We can also use the activity of these chemicals as a reference in the further glioma-related research based on the above theory. Not only novel targets of chemotherapy for glioma patients, but also a series of biomarkers to characterize glioma including serum testosterone, AR, SVIP, PSA, and genotype of p53 could be clinically applied in the future. However, one obstacle in glioma chemotherapy is the blood brain barrier (BBB). Only two studies reported that ARN-509 could permeabilize the BBB [13, 35]. Thus, we aspire to test the uptake of ARN-509 in an intracranial xenograft model in the future investigations.

In summary, we have found that androgens function to promote glioma cell proliferation through activating AR signaling. Then, an AR response gene *SVIP* was negatively regulated on the transcriptional level. Despite AR-p53 mutual suppression, decreased SVIP expression may lead to declined p53^wt^ expression and facilitation of the malignancy progression that could be delayed by AR antagonists. Our results indicate that the role of AR and SVIP in the pathogenesis of different types (subtypes) of gliomas requires being defined discretely.

## MATERIALS AND METHODS

### Antibodies and siRNA

The following primary antibodies were used: polyclonal anti-SVIP, monoclonal anti-NeuN (Millipore), monoclonal anti-CNS (Millipore), monoclonal anti-GFAP (Beyotime Bio, Shanghai, China), and monoclonal anti-CD11b/integrin am (OX-42). Polyclonal anti-β-actin, polyclonal anti-p53, polyclonal anti-Ki67, polyclonal anti-PSA (A67-B/E3), and anti-AR (N-20) antibodies were obtained from Santa Cruz (TX, USA). Control siRNA and siRNA targeting human SVIP have been previously described [36], siRNAs targeting SVIP (5′→3′): siRNA #1, GACAAAAAGAGGCTGCATC; siRNA #2, CCCTTA AGTGCAATGCTAA; siRNA #3, GACAGTTTCATAA AGCATA.

### Cell culture and transfection

HeLa, SKBR-3, U251MG, and U87MG cells were obtained from China Center for Type Culture Collection (CCTCC, Shanghai, China). Neuro2A, SH-SY5Y, and BIU-87 cells were kind gifts from Dr. Yuxian Shen (Anhui Medical University, Anhui, China); SW480 and SW620 cells were gifts from Dr. Siying Wang (Anhui Medical University); PC-3 and LNCaP cells were gifts from Dr. Xuemei Huang (Harbin Institute of Technology, Heilongjiang, China). The cells were cultured in DMEM or RPMI 1640 (Gibco, NY, USA) supplemented with 10% fetal bovine serum (Invitrogen), 100U/ml penicillin, and 100 mg/ml streptomycin in a humidified incubator with 5% CO_2_ at 37°C. For treatment with R1881 or ARN-509 and MDV3100 (Enzalutamide, brand name Xtandi) both obtained from APExBio, TX, USA, cells were grown in phenol red-free DMEM (Gibco) medium supplemented with 10% (or indicated concentration, v/v) dextran-coated charcoal stripped FBS (Biological Industries, Israel) for 72 h prior to treatment. Every 24 h, the medium was replaced with medium with or without hormone and the cells were incubated for a further 24 to 72 h before analysis. Plasmids expressing wild-type SVIP has been previously described [18]. For transient transfection or RNA interference (RNAi), plasmid or siRNA was transfected using Lipofectamine 2000 (Invitrogen) according to the manufacturer's instructions.

### Immunoblotting and Immunohistochemistry

Cell lysis, protein extraction, and immunoblotting were performed as described previously [18]. The formalin-fixed-paraffin-embedded tissue sections (4 μm) were processed according to the manufacturer's instructions (Histostain Plus Kit SP-9000, Zymed, CA, USA). The dilution and incubation with antibodies were as fowllowed: anti-AR 1:500, anti-SVIP 1:500, anti-p53 1:250, anti-PSA 1:250, and anti-Ki67 1:250. The sections were then counterstained with hematoxylin.

### Immunofluorescence

Cells or cryostat sections (5 mm thick) of rat brain tissue were fixed with 4% paraformaldehyde in PBS at 4°C for 30 min and then subjected to immunofluorescence staining as described previously [18]. Cells or cryostat sections were incubated with anti-AR (N-20) pAb (1:250), anti-NeuN mAb (1:200), anti-CNS mAb (1:200), anti-GFAP mAb (1:200), or anti-CD11b/integrin am (OX-42) mAb (1:200), respectively.

### Cell proliferation assay

72 h after RNA transfection or indicated treatment, the following assays were performed. For MTT assay, cells were seeded into 96-well plates at a density of 4000 cells/well. After treatment, the medium was replaced with fresh culture medium contained 0.5 mg/ml MTT reagent and incubated at 37°C for 4 h. Subsequently, the supernatant was aspirated, and cells were lysed in 200 μl DMSO for 10 min at 37°C. The optical density (OD) was measured at 490 nm using a plate reader. For cell count, the cells were split into 24-well plates. 5 images of the cells were captured on 5 random view fields from every well of a 24-well plate, by microscopy. Then, the cell numbers were estimated by Cell Profiler (www.cellprofiler.org), and an average of the 5 view fields was presented. Soft agar assay was carried out as a classic protocol on a 6-well plate with 1000 cells/well. After 16 days, the colonies were stained with 0.005% crystal violet and observed under a microscope. Each experiment was performed in triplicate.

### Apoptosis detection by flow cytometry

Apoptotic cells were differentiated from viable or necrotic cells by combined application of Annexin V-FITC and propidium iodide (PI) (BD Biosciences Clontech, USA). The samples were washed twice and adjusted to a concentration of 1 × 10^6^ cells/ml with cold PBS. 100 μl of the cell suspension was mixed with 10 μl of annexin V-FITC and 10 μl PI (20 μg/ml) in a tube and incubated for at least 20 min at room temperature in the dark. Subsequently, 400 μl of PBS binding buffer was added to each tube without washing and analyzed immediately by flow cytometry analysis (BD Biosciences). This assay was performed in triplicate.

### Tissue sample collection

The formalin-fixed-paraffin-embedded tissue samples, including 73 glioma tissues and 12 normal brain tissues, were obtained from the patients who underwent surgical treatment from July 2011 to July 2013. Glioma tissue samples were obtained from patients with primary gliomas according to 2007 WHO classification [37], 8, 15, 23, and 27 cases were classified as grade I, II, III, and IV, respectively. Normal brain tissues as normal controls were obtained from the patients with severe traumatic brain injury (TBI) who underwent decompression surgery. None of the patients had received chemotherapy, immunotherapy, and radiotherapy prior to specimen collection.

### Serum sample collection

Serum samples were collected from three groups, glioma group, benign brain tumor group, and healthy individuals. The glioma group consisted of 122 male and 67 female patients with a median age of 49 years (range, 6, 75 years), different from the source of tissue samples, with histologically confirmed glioma who underwent surgical treatment. The benign brain tumor group consisted of 64 male and 112 female patients with a median age of 47 years (range,12, 75 years), 176 patients who underwent surgery and were histologically confirmed with pituitary adenoma (*n* = 71), benign meningioma (*n* = 83), and acoustic neuroma (*n* = 24). The normal control group consisted of 118 individuals with functional nervous disorders but not benign or malignant tumors and comprised of 42 males and 76 females with a median age of 51 years (range, 33, 71 years). Young patients (< 30 years) with functional nervous disorders were rare and were not included in the normal control group.

### Conventional reverse transcriptional PCR

Total RNA was extracted from control and SVIP knockdown HeLa and SKBR3 cells, R1881 treated, and control U87MG or U251MG cells using TRIzol reagent (Invitrogen) following the manufacturer's instructions. Reverse-transcriptional PCR was performed as described previously for 25 and 30 cycles in order to reduce the effect of amplification saturation [18]. The primers are described in the Supplementary materials. This assay was performed in triplicate.

### Mouse xenograft models

Four weeks old male BALB/C nude mice, obtained, and maintained in specific pathogen free Animal Center of Dalian Medical University, were castrated. 1week after castration, mice were subcutaneously injected in the flank with 5 × 10^6^ U87MG cells mixed with 100 μl PBS. The tumor size was measured daily with a caliper beginning 1 week after inoculation, and the volume was calculated as π/6 × length × width^2^. After the tumor volume had reached 100 mm^3^, the mouse was treated with MDV3100 (APExBio) or placebo (control) at a dose of 10 mg/kg body weight by daily oral gavage. MDV3100 stock were prepared in vehicle aqueous slurry (placebo), containing 20% PEG-400, 0.25% Tween-80, and 0.5% w/v carboxymethylcellulose (CMC) solution in 20 mM citrate buffer (pH 4.0), stored at 4°C. Xenograft tumors were excised upon sacrifice on day 14 post-drug treatment.

### *Ex vivo* optical imaging

Mice bearing human U87MG tumor xenografts were injected intravenously with IR-780 iodide (Sigma) at a dose of 0.2 mg/kg. Whole-body optical images were captured 72 h after dye injection using a Kodak *In-Vivo* FX Professional Imaging System equipped with fluorescent filter sets (excitation/emission, 770/830 nm). The field of view was 120 mm in diameter. The frequency rate for NIR excitation light was 2 mW/cm^2^. Prior to imaging, mice were anesthetized by intra-abdominal injection of 1% pentobarbital sodium (45 mg/kg).

### Ethics approval

The study was performed in accordance with the 1964 Helsinki Declaration. Tissue and serum sample collection were approved by the ethics committee of Anhui Provincial Hospital Affiliated to Anhui Medial University and written informed consent was obtained from all patients. All mice experiments were approved by the Dalian Medical University Animal Care and Use Committee.

### Statistical analysis

SPSS 17.0 was used for statistical analysis in the present study. Ranked data analysis was performed using rank-sum test. Measured data were reported as means ± SD and analyzed using one-way analysis of variance (ANOVA) with Bonferroni's post hoc analysis, respectively. Chi-squared test or Fisher's exact test was used to assess the correlation between the expression of AR and the clinicopathological features. *P* < 0.05 was considered statistically significant.

## SUPPLEMENTARY MATERIALS FIGURES



## Abbreviations

AR: androgen receptor
BBB: blood brain barrier
CMC: carboxymethylcellulose
CRPC: castration- resistant prostate cancer
ER: endoplasmic reticulum
ERAD: endoplasmic reticulum-associated degradation
GBM: glioblastoma multiforme
IHC: immunohistochemistry
MTT: methylthiazolyldiphenyl-tetrazolium bromide
OD: optical density
PI: propidium iodide
PSA: prostate-specific antigen
SVIP: small VCP/p97-interacting protein
TBI: traumatic brain injury
TMZ: temozolomide
WHO: World Health Organization
