# Supplementary material for: Regulation of p53wt glioma cell proliferation by androgen receptor-mediated inhibition of small VCP/p97-interacting protein expression
Source: Oncotarget. 2017 Feb 19;8(14):23142–54. doi: 10.18632/oncotarget.15509 (PMC5410292; doi:10.18632/oncotarget.15509)
Supplement: Supplementary file 1 [file oncotarget-08-23142-s001.pdf]

# Regulation of p53[wt] glioma cell proliferation by androgen receptor-mediated inhibition of small VCP/p97-interacting protein expression

**Supplementary Materials**

## MATERIALS AND METHODS

### Conventional RT-PCR primers

| Target | Sequence 5′→3′ |
|---|---|
| SVIP | Forward: GTCAGGGTTCTCAAGCTGTCG |
| | Reverse: GTCTAGCCATTCTCTAAGCTTG |
| AR full lenth | Forward: CTACTCCGGACCTTACGGGGACATGCG |
| | Reverse: GGGCTGACATTCATAGCCTTCAATGTGTGAC |
| AR V7 | Forward: CTACTCCGGACCTTACGGGGACATGCG |
| | Reverse: TGCCAACCCGGAATTTTTCTCCC |
| β-actin | Forward: TGTTTGAGACCTTCAACACC |
| | Reverse: ACGCAGGATGGCATGG |
| p53 | Forward: ATGGAGGAGCCGCAGTCAGA |
| | Reverse: ACATCTTGTTGAGGGCAGGG |

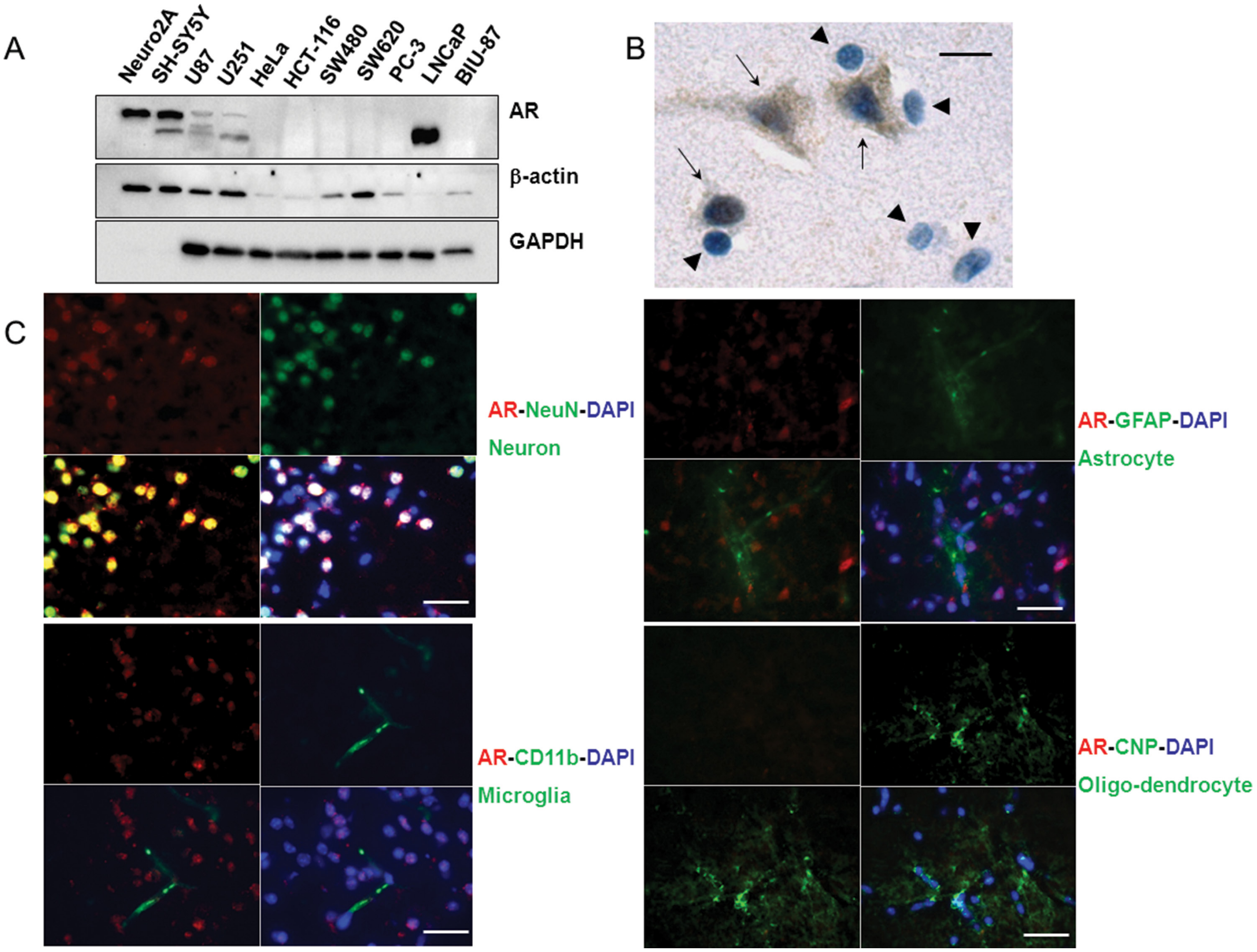


**Supplementary Figure 1: Androgen receptor is highly expressed in neuron and glioma cell lines, but not in glial cells of normal brain tissue.** (**A**) Whole-cell lysates were subjected to western blotting assay for AR expression in neuroblastoma cell lines Neuro2A and SH-SY5Y, human glioblastoma cell lines U87 and U251, cervical cancer cell HeLa, colon cancer cell lines SW480, SW620, and HCT-116, prostate cancer cell lines LNCaP and PC-3, and bladder cancer cells BIU-87. GAPDH and β-actin were used as loading control. (**B**) IHC assay of a paraffin-embedded tissue section of mouse brain using anti-AR antibody (N-20) at a concentration of 1:500, AR expressed in neuron (indicated by arrow) of normal mouse brain tissue, but not in glial cells (indicated by arrowhead), scale bar = 50 µm. (**C**) AR tagged with TRITC is expressed in the neurons, immunofluorescence assay of AR expression in rat brain tissues, 100×, scale bar = 100 µm. Neurons, astrocytes, microglias, and oligodendrocytes were marked by NeuN, GFAP, integrin αm (OX-42) and CNP, tagged with FITC, respectively. The nuclei of the cells were stained with DAPI.

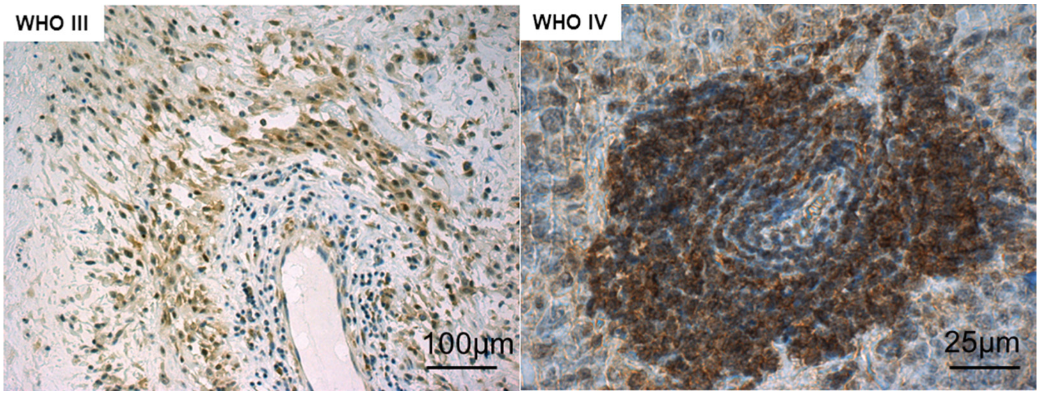


**Supplementary Figure 2: Extremely high expression of AR, analyzed by IHC, around the vessels on high-grade glioma specimens.** Left panel, WHO III, astroglioma; right panel, WHO IV, glioblastoma.

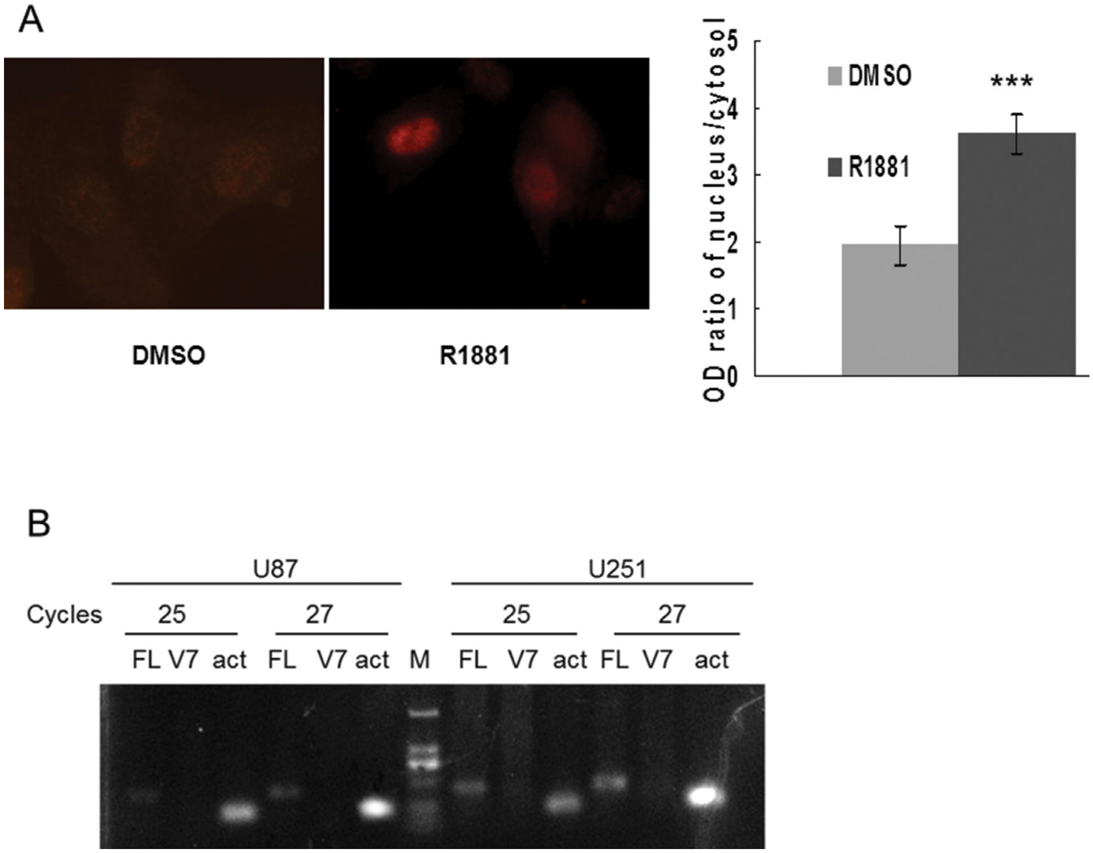


**Supplementary Figure 3:** (**A**) AR relocated to the nuclei of U87 cells with 10nM R1881 treatment for 24h. The optical density of nuclear to the cytosolic area was analyzed in 10-20 cells/field of 5 random view fields. ***$P$ < 0.01. (**B**) Semi-quantitative PCR analysis of full length AR and splice varient AR-V7 mRNA level in U87 and U251 cell lines. FL, full length AR; V7, splice varient AR-V7; act, actin as loading control; M, DNA marker.

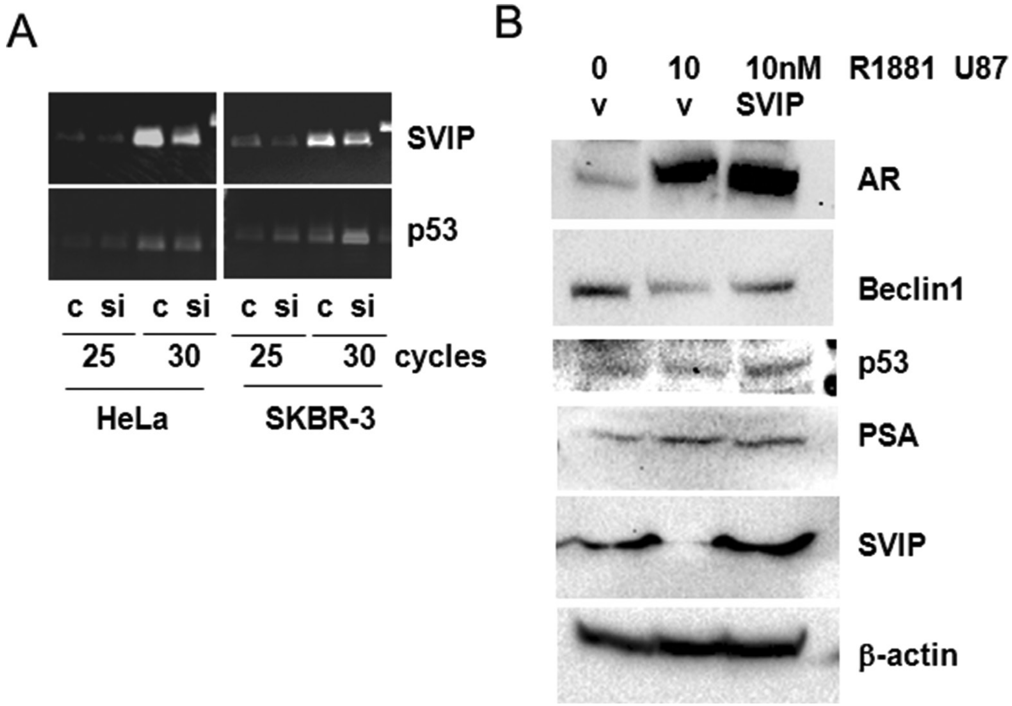


**Supplementary Figure 4: SVIP regulates p53 on post-transcriptional level.** (**A**) mRNA level of p53 was not associated to SVIP knockdown by siRNA, as analyzed by reverse transcriptional PCR. 25 and 30 cycles of PCR were performed to reduce the effect of amplification saturation. (**B**) p53 rescued by exogenous SVIP expression. U87 cells were pretreated with DMSO or 10 nM R1881. 48 hours after R1881 treatment, cells were transfected with vector (v) or SVIP-expression plasmid. 24 hous after transfection, cells were lysed and subjected to Western blotting assay. β-actin was used as a loading control.
